# Pancreatic islet transplantation

**DOI:** 10.1186/1758-5996-1-9

**Published:** 2009-09-21

**Authors:** Maria Lúcia Corrêa-Giannella, Alexandre S Raposo do Amaral

**Affiliations:** 1 Laboratory of Cellular and Molecular Endocrinology (LIM25) - University of São Paulo Medical School, São Paulo, Brazil

## Abstract

**Background:**

No formulation of exogenous insulin available to date has yet been able to mimic the physiological nictemeral rhythms of this hormone, and despite all engineering advancements, the theoretical proposal of developing a mechanical replacement for pancreatic β cell still has not been reached. Thus, the replacement of β cells through pancreas and pancreatic islet transplantation are the only concrete alternatives for re-establishing the endogenous insulin secretion in type 1 diabetic patients. Since only 1 to 1.5% of the pancreatic mass corresponds to endocrine tissue, pancreatic islets transplantation arises as a natural alternative. Data from the *International Islet Transplant Registry *(ITR) from 1983 to December 2000 document a total of 493 transplants performed around the world, with progressively worse rates of post-transplant insulin independence. In 2000, the "Edmonton Protocol" introduced several modifications to the transplantation procedure, such as the use of a steroid-free immunosuppression regimen and transplantation of a mean islet mass of 11,000 islet equivalents per kilogram, which significantly improved 1-year outcomes. Although the results of a 5-year follow-up in 65 patients demonstrated improvement in glycemic instability in a significant portion of them, only 7.5% of the patients have reached insulin independence, indicating the need of further advances in the preservation of the function of transplanted islet. In addition to the scarcity of organs available for transplantation, islets transplantation still faces major challenges, specially those related to cell loss during the process of islet isolation and the losses related to the graft site, apoptosis, allorejection, autoimmunity, and immunosuppression. The main strategies to optimize islet transplantation aim at improving all these aspects.

**Conclusion:**

Human islet transplantation should be regarded as an intervention that can decrease the frequency of severe hypoglycemic episodes and improve glycemic control in selected patient for whom benefits of 4-5 years duration would be very valuable. Its limitations, however, indicate that the procedure in its current format is not suitable for all patients with type 1 diabetes.

## Introduction

Chronic complications related to Type 1 Diabetes are closely associated to the onset and maintenance of hyperglycemia, as demonstrated by the Diabetes Control and Complication Trial (DCCT) [1] and its follow-up study (EDIC) [2]. No formulation of exogenous insulin available to date has yet been able to mimic the physiological nictemeral rhythms of this hormone. Despite all engineering advancements, the theoretical proposal of developing a mechanical replacement for pancreatic β cell, which should carry a glucose sensor attached to an insulin pump capable of performing real time interpretation of glucose variations, as well as allied and sustained automatic corrections with feedback, still has not been reached.

### Replacement Therapy

To date, the replacement of β cells through pancreas and pancreatic islet transplantation are the only concrete alternatives for re-establishing the endogenous insulin secretion in type 1 diabetic patients. Both procedures have as a barrier the major initial obstacle to all transplants: organ scarcity [3]. The vast majority of pancreas and pancreatic islet transplantation is performed with deceased-donor organs: brain death or, more recently, following cardiac arrest [4]. The search for new sources of cells for implants, by differentiation of embryonic or somatic cells is still hindered by ethical and technical problems. Improvements in organ preservation techniques, surgical transplantation techniques, immunosuppression, rejection diagnosis and management of post-procedure complications have led to considerable progress in the overall survival of grafts and patients [5].

Despite the promotion of good glycemic control and the improvement of the recipient's quality of life, the solitary pancreas transplant may increase the immediate risk of death mainly due to the complexity of the surgery. Moreover, the presence of exocrine tissue seems to enhance immunogenicity and, thereby, promote rejection [5].

Since only 1 to 1.5% of the pancreatic mass corresponds to endocrine tissue and because the exocrine tissue produces a strong immune response, the transplantation of pancreatic islets arises as a natural alternative for re-establishing endogenous insulin secretion. The first report dates from 1893: a xenotransplant with fragments from the pancreas of a lamb [6]. It was only in 1967 that Lacy was able to revert diabetes in rats with transplantation of pancreatic islets [7]. Nonetheless, subsequent results of clinical trials have brought frustrating outcomes.

Data from the *International Islet Transplant Registry *(ITR) [8] from 1983 to December 2000 document a total of 493 transplants performed around the world, with progressively worse rates of post-transplant insulin independence: 66% after 1 month, 40% after 1 year, 22% after 2 years, 11% after 3 years, 6% after 4 years and only 2% after 5 years. However, the quality of the transplants has been improving throughout the years. Of a total of 237 ITR-registered transplants performed between 1990 and 1999, the one-year survival rates for patient and graft (defined as baseline C-peptide > 0.5 ng/mL) were 96% and 41%, respectively. However, the proportion of insulin-free patients at one year was only 11%. The transplantation of over 6,000 islet-equivalents (IE, the number of islets normalized to an islet of 150 μm of diameter) per kg, less than 8 hours of cold ischemia time and a immunosuppression regimen containing anti-T cell antibodies have elicited higher rates of insulin independence. Until then, most islet transplantations were performed simultaneously or after a kidney transplant [8], in patients who therefore presented a very poor metabolic control and progression of vascular complications.

In 2000, the group at University of Alberta, in Edmonton [9] published a series of 7 patients submitted to pancreatic islet transplantation in whom the outcomes were superior to those obtained so far. Patients were 44 years old on average (ranging from 29 to 54 years old), with a mean diabetes duration of 35 years (ranging from 18 to 50 years), and normal kidney function. The inclusion criteria were: type 1 diabetes for over 5 years, C-peptide < 0.48 mg/mL, poor glycemic control despite intensive insulin therapy, recurrent severe hypoglycemia with coma or metabolic instability, to whom the risks of transplantation and immunosuppression were considered to be lower than the risks associated to brittle diabetes. The "Edmonton Protocol", as it is known, introduced several modifications to the transplantation procedure, and the most important were: [1] induction with anti-IL2-receptor antibodies; [2] steroid-free immunosuppression regimen with the use of sirolimus and tacrolimus; [3] transplantation of islets that were freshly prepared without xenoproteins; [4] transplantation of a mean islet mass of 11,000 IE/kg. All recipients required islets from at least two donor pancreases to achieve sustained insulin independence after a median follow-up of 11.9 months.

In 2005, the same group reported the outcomes of a 5-year follow-up in 65 patients submitted to islet transplantation. The median duration of insulin independence was 15 months and although the majority of patients (82%) presented graft survival (C-peptide positivity), only 7.5% maintained insulin independence after 5 years. The glycemic control evaluation demonstrated that patients who were back on insulin, but maintained a positive C- peptide benefited from the transplantation, as shown by an HbA_1_C of 6.7%, while patients who have lost graft function presented an HbA_1_C of 9.0% [10].

These findings can be considered promising, since improvement in glycemic instability was observed in a significant portion of patients, but it has reinforced the question of whether islet transplantation should be aimed at restoring insulin independence or providing adequate metabolic control. The GRAGIL2 trial was a phase 1-2 study designed to address this issue: primary and secondary endpoints were, respectively, the rate of insulin independence and the success rate (defined by a composite score based upon basal C-peptide, HbA_1_C, hypoglycemic events, and exogenous insulin needs). Ten type 1 brittle diabetic patients experiencing severe hypoglycemia received 11,089 ± 505 IE/kg, a maximum of two islet infusions and a immunosuppression regimen similar to the one employed by the Edmonton Protocol. At 12 months, insulin independence was observed in 3 of 10 patients and success in 5 of 10 patients, demonstrating that islet transplantation failed to promote insulin independence, but tended to succeed in improving metabolic control [11].

Another trial which reported similar results was the one designed to test the reproducibility of the Edmonton Protocol, organized by the Immune Tolerance Network and which involved 36 patients in 9 islet transplantation centers. Only 5 (13.8%) patients sustained insulin independence after two years, but subjects with partial graft function had a substantial benefit in the metabolic control. As anticipated, the achievement of insulin independence after 1 year was significantly influenced by the previous experience at each center, suggesting that regionalization of islet-processing facilities could reduce the variation in outcome and improve efficiency in future trials [12].

The evaluation of the impact of islet transplantation on chronic complications is hampered by the uncontrolled and retrospective nature of most of the studies, but it seems that restoration of islet function protects against long-term diabetic complications [13]. Várkonyi et al. followed 11 patients with a mean duration of functioning fetal pancreatic islet graft of 9.5 ± 0.2 years and demonstrated interruption of progression of all microangiopathic complications [14]. Fiorina et al. studied the renal function of 36 patients submitted to combined kidney islet transplantation, comparing patients with successful (fasting C-peptide > 0.5 ng/mL for >1 year) *versus *unsuccessful islet transplants (fasting C-peptide < 0.5 ng/mL). The successful group demonstrated improvements in renal function and better kidney graft survival rates after 7 years of follow-up [15]. In another study, a successful combined kidney islet transplantation was associated with improvement of cardiovascular function (ejection fraction, peak filling rate in end-diastolic volume per second, QT dispersion) for up to 3 years of follow-up compared with the group who experienced an early failure of the islet graft or did not receive an islet graft [16].

Stabilization of diabetic retinopathy after functioning islet transplantation has been demonstrated in a series of 12 patients followed for up to two years [17] and a recent study reported an increase in the retinal arterial and venous blood flow at one year in 10 patients submitted to islet transplantation suggesting a beneficial effect of the metabolic control on retinal microcirculation [18]. A positive effect on neuropathy has also been observed after functioning islet transplantation, with stabilization or even improvement of nerve conduction velocities [17,19].

Considering the negative impact of brittle diabetes on the quality of life (QoL), especially in those patients experiencing severe hypoglycemic episodes, QoL analyses is an important endpoint of clinical trials. While some authors demonstrated that islet transplantation significantly reduces fear of hypoglycemia but have little impact on health-related overall QoL [20], others identified a significant improvement with the use of a diabetes-specific questionnaire (DQoL). In this latter study, the most common reported beneficial effects were glycemic stability and absence of hypoglycemia resulting in a feeling of independence not experienced before the transplant [21].

### Difficulties Related to Pancreatic Islet Transplantation

In addition to the scarcity of organs available for transplantation, pancreatic islets transplantation still faces major challenges, specially those related to cell loss during the process of islet isolation and the losses related to the graft site, apoptosis, allorejection, immunosuppression, and autoimmunity. The main strategies to optimize pancreatic islet transplantation aim at improving all these aspects.

### Related to the donor and to the process of islet isolation

Even with the use of selection criteria to define the best pancreas donors and avoid allocation of inadequate organs for transplant, islets start experiencing damage before pancreas removal because brain death is related to the production of pro-inflammatory cytokines like TNF, IL-1beta and IL-6 which induce cell death and decrease the quality of the graft [22]. Donor characteristics such as age, previous medical history, body mass index, cause of death, use of vasopressive agents, and pancreatic size and fat content are factors that influence the quality of isolated islets [23,24].

Donor age seems to be directly linked to the quality of the isolated islets. In younger donors, under 20 years old, it can be difficult to isolate and purify islets without promoting islet fragmentation due to over digestion. In donors over 50 years of age, on the other hand, pancreatic digestion is facilitated - however, the functional reserve of the islets may be reduced [23]. Ischemia-reperfusion is an important mechanism of cellular injury in transplants in general. Since pancreatic islets require a substantial blood supply in order to remain viable [25], ischemia causes great damage to the pancreatic tissue. Different preservation methods and solutions aim at reducing the deleterious effects of tissue hypoxia, the main being hypothermia and intra-pancreatic infusion of UW's (*University of Wisconsin*) preservation solution, which contains glucose, electrolytes, colloid, glutathione, allopurinol, adenosine, and other metabolic substrates which preserve cellular integrity. Although UW's solution can preserve the pancreas for over 24 hours, the longer the period, the worse the quality of the graft. More recently, the combined use of UW and compounds with high oxygen affinity (two-layer method) has optimized organ preservation [26].

The isolation of pancreatic islets is comprised of several steps. After removal of the donor's pancreas, the islets are maintained in the preservation solution until the beginning of the isolation process. Next, a solution of purified collagenase is infused in the pancreatic duct and the pancreas is cut and placed in a chamber at 98.6°F. There is a period of shaken with the use of small spheres of metal in order to promote tissue dissociation through enzymatic and mechanical digestion. The final separation of the exocrine and endocrine tissues is performed by Ficoll density gradient. The human islet preparations are evaluated regarding purity, morphologic and functional viability (insulin secretion) and content of endotoxin. The number of purified islets is expressed as the number of IE.

The functions of the endocrine pancreas are closely related to its microarchitecture. Interactions with acinar and mesenchymal tissues are essential ever since the embryonic morphogenesis up to the development of mature secretory cells [27]. The metabolic control depends not only on the integrity of the islets, but also on the interaction among beta and non-beta cells within the pancreatic islet, as well as with surrounding islets [28,29]. Therefore, the function and viability of pancreatic islets are compromised by the separation of the exocrine tissue which precedes the cell transplant. The mechanical trauma and the exposure to collagenase and to pancreatic enzymes fragment islets, damaging especially the non-beta cells located at the periphery [30]. Additionally, the complex vascular and neuronal network of the pancreas is destroyed, significantly modifying the biochemical communication among cells. Thus, the process of islets isolation results in pro-inflammatory and oxidative stress states, with loss of cellular viability and apoptosis [31] which begin soon after the isolation and are closely associated to the failure of the islet transplant [32].

Although it confers flexibility to the transplant, islet culture seems to be related to loss of cellular mass. One of the key points of the Edmonton Protocol was the transplant of freshly isolated islets, however, the same group has recently published a study with human islets placed in culture before transplantation. Although a reduction in the amount of cells had been observed, there was an improvement in islets morphology and viability [33].

### Related to the recipient

In pancreas transplantations, an increase in the concentrations of anti-GAD (Glutamic Acid Decarboxylase) and anti-IA2 (protein tyrosine phosphatase, IA-2) autoantibodies is related to the onset of rejection or graft loss [34]. In islet transplantation, the presence of autoantibodies also correlates with a worse evolution and could be a key factor in the chronic failure of the graft [35].

Recipient's characteristics also influence the outcome of islet transplantation. Eligible candidates should be between 18 and 65 years of age, have type 1 diabetes for more than 5 years with undetectable serum concentrations of C-peptide, and present recurrent episodes of hypoglycemia, usually associated with hypoglycemia unawareness, labile diabetes or progressive chronic complications. In the Immune Tolerance Network. [12], advanced, non-treatable coronary arterial disease; body mass index > 26 kg/m^2^; insulin requirements > 0.7 UI/kg; HbA_1_C > 12%; serum creatinine > 1.5 mg/dL or creatinine clearance < 80 mL/min/1.73 m^2 ^and albuminuria > 300 mg/24 h, active infections, severe psychiatric diseases or conditions that may hinder comprehension and treatment compliance were considered exclusion criteria.

Acute complications are related to the procedure itself - bleedings, branch portal vein thrombosis after islet infusion, accidental gallbladder puncture and transient increases of transaminases, while chronic complications are related to the immunosuppressive regimen and graft rejection. [10]

The implant site also contributes to islets loss. Although the subcapsular renal space is the site most commonly used in animal models because it allows graft recovery, the liver is the site of choice for islet transplantation in humans. Access can be obtained through laparotomy or more commonly, through portal vein cannulation monitored by radioscopy [36]. It is believed that the portal blood enriched in growth factors, the physiological actions of insulin and glucagon on hepatic glucose metabolism, and the easy access to portal vein would be favorable factors. On the other hand, hypoxic injury to the presinusoidal hepatocytes after islet embolization and an inflammatory reaction ultimately leading to islet apoptosis might take place [37]. Also, focal hyperinsulinemia and its correlation with hepatic steatosis [38], the direct action of immunosuppressants on islets [39] before the first hepatic pass and difficulties in performing biopsies during the follow-up would be negative factors to the short-term survival of the islets at this site. After infusion, the islets are prone to developing nonspecific inflammatory responses to HLA, with the activation of coagulation and complement systems. Besides, immediately after transplantation, islets are dependent upon diffusion of oxygen and nutrients until revascularization by angiogenesis is established, a process that takes 7-10 days. This hostile environment with low oxygen tension, increased production of oxygen-reactive species and presence of inflammatory cytokines is plenty of stimuli to short-term cell death. Thus, in the first few days following transplantation, even under excellent metabolic control, the grafted tissue is exposed to unfavorable conditions that may result in cell death [40]. It is estimated that 50-70% of islets are destroyed in the immediate post transplant period [41]. However, after three months, the islets are surrounded by endothelial tissue, being nourished by a rich capillary network, as demonstrated in a nonhuman primate model of allogeneic transplantation [42].

Evaluating β-cell function after islet transplantation is a difficult task. β-Score is a proposed scoring system determined from plasma glucose values, HbA1c, daily insulin requirement and stimulated C-peptide response. This measurement may be more useful than simply assessing the presence or absence of insulin independence [43]. Recently, Caumo et al. described a simpler and cheaper score named Transplant estimated function (TEF), which takes into account only daily insulin requirement and HbA1c and that can be normalized to the number of transplanted islets [44]. Radiological methods to detect graft damage prior to metabolic impairment have also been sought. Magnetic resonance imaging monitoring after transplantation of iron-labeled islets was shown to be feasible and safe in the clinical practice, but this technique needs optimization, such as improvements on image resolution and development of quantification methods to better correlate the signal with the islet mass [45].

The lack of specific clinical markers of allograft rejection poses another challenge, since current methods rely only on monitoring glucose homoeostasis, which may not be disturbed during the earliest stages of rejection. Also, the nature of islet transplantation does not permit a biopsy approach. Thus, the development of a non-invasive clinical assay for detection of early rejection is a very important step to allow early intervention to reduce the risk of graft loss [32].

ABO compatibility and a negative donor-recipient serum cross-match for T cells are required for islet transplantation, while HLA antigen matching is not. [46]. The immunosuppressive regimen used in the Edmonton Protocol, a steroid-free combination therapy including dacluzimab, sirolimus and tacrolimus has improved transplantation outcomes. However, tacrolimus has diabetogenic effects [47] and there are *in vitro *studies showing a direct deleterious effect of sirolimus on human islets, impairing glucose-stimulated insulin secretion. On the other hand, there are experimental and clinical data indicating that sirolimus exerts a beneficial role in the early post-transplant inflammatory phenomena and some authors suggest that the effect of sirolimus may vary from helpful to harmful depending on the circumstances and that tailoring immunosuppression might apply to islet transplantation with regard to the use or avoidance of this immunossupressor [48].

Additionally, both tacrolimus and sirolimus are able to interfere with β cell regeneration [49] and a study with type 1 diabetic patients who received the immunosuppressive drugs used in the Edmonton Protocol demonstrated that the loss of T-cells associated to this regimen determined an increase in serum concentrations of IL-7 and IL-15, and the proliferation of CD45RO+ memory T-cells, enriched in specific autoreactive T-cell clones for GAD 65, which could contribute to the recurrence of autoimmunity in these patients [37]. These findings suggest that modifying the immunosuppression regimen of the Edmonton protocol might have positive effects on graft survival and function.

Islet transplantation has brought better outcomes and reached standardization to a certain extent only after the Edmonton results in 2000, but benefits of islet transplantation still remain to be determined.

## Perspectives

Among the main measures necessary to achieve better long-term results in islet transplantation is the development of new immunosuppressive agents and of strategies to induce immunological tolerance. The increase in the number of successful islets transplantations also require the elimination of the need for multiple islet donors per recipient [46].

Several attempts to increase the viability of transplantable islets have been proposed, mainly related to improvement of islet isolation process, to pharmacological interventions and to new immunosuppressive protocols. Many interventions are being tested to improve islet survival, such as the use of growth factors [50], glucagon-like peptides (GLP) and GLP-1 analogs [51], oxygen carriers [52], substances that reduce inflammatory response [53], anticoagulants [54], antiapoptotic drugs [55] and antioxidant compounds [44]. Immunoisolation of transplanted islets with semi-permeable membranes is an attractive strategy to preclude the use of immunosuppressive agents, however, the perfect immunoisolation system has not been found yet.

Prolonged reversal of diabetes obtained in non-human immunosuppressed primates transplanted with porcine pancreatic islets suggests that xenotransplant with pig islets may be an alternative to circumvent problems of islet availability for diabetic patients. However, for this transplant modality to reach clinical applicability, long-term clinical trials should be performed. Among the aspects to be specifically examined are overcoming the immune reaction and carefully evaluating the risk of porcine endogenous retrovirus (PERV) transmission [45]. This last aspect is extremely relevant, considering that recipients of xenografts might be taking immunosuppressive drugs, which decrease the host resistance, increasing the possibility of virus (re)activation. In spite of these safety issues, some researches believe that, in the future, it will be possible to move forward from pre-clinical studies into clinical trials of xenotransplantation [46].

One of the major limitations of islet transplantation is the scarcity of islets. Therefore, strategies to develop islet cells *in vitro *have been viewed as an alternative to increase β cell availability prior to transplantation. Recently published data provided convincing evidence that human embryonic stem cells (hES) are competent to generate glucose-responsive, insulin-secreting cells [56]. However, besides ethical controversies, safety issues have to be addressed adequately to ensure that potentially tumorigenic hES are not transplanted among differentiated islet cells. The use of multipotent stem cells present within the adult pancreas or the transdifferentiation of exocrine acinar and/or ductal cells into β cells may circumvent these drawbacks, but it still faces some obstacles such as uncertainty as to the exact nature of stimuli necessary to promote complete differentiation and the low yield of the *in vitro *differentiation procedure.

In conclusion, human islet transplantation should be regarded as an intervention that can decrease the frequency of severe hypoglycemic episodes and improve glycemic control in selected patient for whom benefits of 4-5 years duration would be very valuable. The limitations that have been realized, however, indicate that the procedure in its current format is not suitable for all patients with type 1 diabetes [57]. Efforts should be made to improve outcomes of this treatment modality, still considered experimental, before it can be designated as "non-research", as it already happens in Canada.

## Competing interests

The authors declare that they have no competing interests.

## Authors' contributions

This manuscript was written by both co-authors; ASRA performed the literature review. Both authors read and approved the final manuscript.
